# Spectroscopic Study on CdS/Ni/KNbO_3_: Confirming Ni Effect to Photocatalytic Activity

**DOI:** 10.1021/acsomega.3c04829

**Published:** 2023-09-11

**Authors:** Su Young Ryu, Tai Kyu Lee, Michael R. Hoffmann

**Affiliations:** † Environmental Science & Engineering, Linde Laboratory, California Institute of Technology, Pasadena, California 91125, United States; ‡ Nanopac Co., Ltd., Giheung-gu, Yongin-si, Gyeonggi-do 17015, Republic of Korea

## Abstract

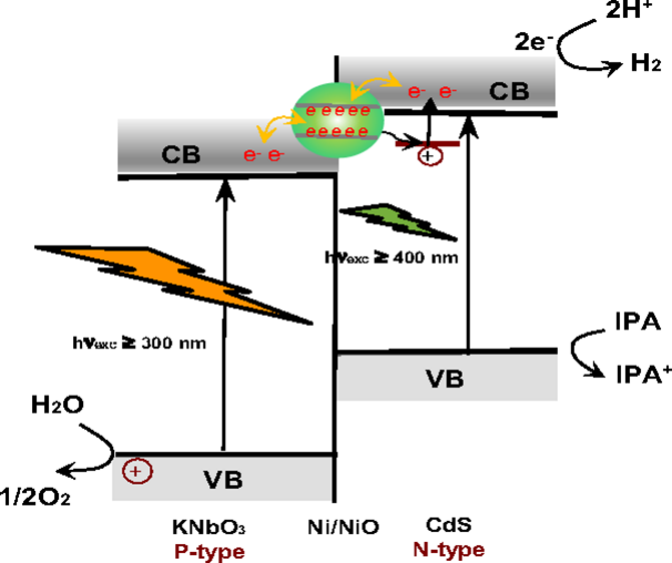

Herein, we report the structural and photophysical properties of CdS/Ni/KNbO_3_ composites with a quantum yield for photocatalytic H_2_ generation that is CdS and Ni amount dependent. The nonstoichiometric KNbO_3_ (1:1.1) structure indicates the defect at the K site, which is Ni-occupied during its deposit process. It exhibits a tendency like a Ni-doped characteristic up to 0.1 wt % Ni and then forms a Ni cluster in case the Ni amount exceeds 0.1 wt %. The related structural and photophysical properties of CdS/Ni/KNbO_3_ are examined with Fourier transform infrared, X-ray diffraction, ultraviolet–visible absorption, and luminescence spectral analysis. It demonstrates the CdS/Ni/KNbO_3_ composites to be an efficient light conversion caused by efficient charge/electron transfer between KNbO_3_ and CdS via doped Ni. The photocatalytic activity of CdS/Ni/KNbO_3_ exhibits a CdS and Ni amount dependency. The best photocatalytic activity for H_2_ generation is obtained with 0.1 wt % Ni and 2.9 wt % CdS as it gradually declines with the excess Ni amount than 0.1 wt % caused by a formed Ni cluster.

## 1 Introduction

Hydrogen production from water using a semiconductor photocatalyst has attracted considerable interest in the conversion of solar energy. Honda and Fujishima discovered that water can be photo-electrochemically decomposed into hydrogen (H_2_) and oxygen (O_2_) using a TiO_2_ electrode under UV light irradiation in 1972.^1^ Since then, many of the metal oxide semiconductors such as Nb_2_O_5_, Ta_2_O_5_, ZrO_2_, SrTiO_3_, SrNb_2_O_7_, and SrTa_2_O_7_, etc. have been discovered to be activity for water splitting into H_2_ and O_2_. Especially, K_4_Nb_6_O_17_ has attracted considerable interest in the redox reaction due to an unique layered structure consisting of two characteristic interlayers (I and II) showing different physical and chemical properties.^2−4^ Indeed, Ni/K_4_Nb_6_O_17_ and Pt/K_4_Nb_6_O_17_ that are intercalated Ni or Pt in the interlayer I as a co-catalyst have been reported to have a great photocatalytic activity for water splitting into H_2_ and O_2_ caused by delocalized charge carriers in the NbO_6_ octahedral structure.^2−5^ However, most metal oxide semiconductors have photocatalytic activity under UV light only (λ ≤ 400 nm, ∼5% of solar light) due to their wide band gap since the valence band mainly consists of O 2p orbitals, whose electrical potential is about 3 eV vs NHE.^6^ Thus, a lot of efforts have been made to develop photocatalysts responding to visible light corresponding ∼42% of solar light.

CdS, an n-type semiconductor with *E*_BG_ = 2.4 eV, has been known as a photocatalyst responding to visible light for H_2_ generation.^7,8^ The photocatalytic activity of CdS can be improved by combining to other semiconductors having a different energy level and band gap, for example, TiO_2_/CdS,^9^ ZnO/CdS, ZnS/CdS,^10,11^ and K_4_Nb_6_O_17_/CdS composites,^7,12,13^ resulted in efficient charge separation as a Z-schematic photocatalysis system. The separated charge carriers can prevent or retard the charge recombination, resulting in improved photocatalytic activity.^8,14,15^

In this study, we reveal the photoinduced electron transfer mechanism on the CdS/Ni/KNbO_3_ composite by a diffuse reflectance infrared Fourier transform (DRIFT), UV–vis absorption, and luminescence spectroscopic study. The electronic band structure of KNbO_3_ has been studied in various aspects of research.^6,16,17^ According to KNbO_3_ structural study, the valence band (VB) of KNbO_3_ is derived primarily from oxygen 2*p* with the contribution of Nb 4*d* to the low and middle parts, while the conduction band (CB) is mostly derived from Nb 4*d* orbitals. The calculations of CB generally agree to predict two separated energy structures of Nb 4*dt*_2g_ and e_g_ due to a lift of degeneracy for the Nb 4*d* orbitals in the octahedral NbO_6_ site. On the other hand, the electron states of potassium, K, are mostly located in the upper part of the conduction band (CB) and the lower part of the valence band (VB). Therefore, the electronic property of KNbO_3_ is mainly dependent on the Nb and O, even though the potassium, K, still indirectly affects the electron environment of Nb and O. The electron density of the valence band (VB) increases when Nb 4*d* states strongly hybridize with the O 2*p* orbital, and this hybridization seems to enhance a ferroelectric distortion. Indeed, the ferroelectric distortion induced by displacement or vacancy of a cation site in the structure leads to change of the net dipole moment or polarization in unit volume.^6,16,17^

For this study, we synthesized KNbO_3_ in a nonstoichiometric mixture of K_2_CO_3_ and Nb_2_O_5_ (1:1.1), which creates a vacancy at K^+^ site and affects the structural and photophysical properties of CdS/Ni/KNbO_3_ composite.^14,15^ The hybrid composites combined an n-type CdS and a p-type KNbO_3_ would be an ideal design not only potential advantage by a band gap engineering but also efficient charge/electron transfer at the p-n junction, occurring charge diffusion in the interface. The Ni located in the interface between KNbO_3_ and CdS takes a crucial role as a route of charge and electron transfer, resulting in a Z-scheme characteristic mechanism for the photocatalytic reaction.^14,15^

## 2 Experimental Section

### 2.1 Preparation of Materials

KNbO_3_ is prepared by conventional solid-state reactions at high temperatures as follows: K_2_CO_3_ (Aldrich) and Nb_2_O_5_ (99.9%, Aldrich) (1:1.1 nonstoichiometric) are mixed and ground in a mortar and then pressed with 4000 psi. The pressed pellet is calcined at 1025 °C in air for 10 h with a heating temperature ramp of 200 °C/h. The Ni/KNbO_3_ is prepared by Ni^2+^ deposit on the KNbO_3_ surface, which suspends KNbO_3_ powder in Ni(NO_3_)_2_ aqueous solution for 1 day. The Ni^2+^/KNbO_3_ is heat-treated at 500 °C for 2 h under a H_2_ atmosphere, followed by additional O_2_ treatment at 200 °C for 1 h in a closed gas circulation system. Ni/KNbO_3_ indicates an immediate color change from green to gray with H_2_ treatment at 500 °C. The composite materials of CdS/KNbO_3_ and CdS/Ni/KNbO_3_ are prepared as follows: 1 g of KNbO_3_ or Ni/KNbO_3_ is stirred in 20 mL of 10 mM cadmium acetate ethanol solution for 24 h; then, sulfurization is carried out by the addition of 20 mL of 10 mM Na_2_S ethanol solution and kept stirring for 7 days at room temperature. The powder was washed with ethanol and distilled water several times and subsequently dried at 130 °C.

### 2.2 Analytical Methods and Instruments

The X-ray diffraction (XRD) pattern is measured with a Philips diffractometer (X’pert Pro) with Cu-Kα radiation. UV–vis diffuse reflectance spectra are recorded on a Shimadzu UV-2101PC with an integrating sphere attachment (Shimadzu ISR-260) using Ba_2_SO_4_ powder as an internal reference. The UV–vis absorption spectrum is evaluated by the Kubelka–Munk function of the diffuse reflectance:
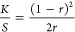
1where *K* and *S* are absorption and scattering coefficients, respectively, and *r* is the diffuse reflectance. The steady-state emission spectrum is measured with a scanning SLM-AMINCO 4800 spectrofluorometer, which enables the corrected spectra by using a Rhodamine B as a quantum counter. The fs-mode locked Ti–sapphire laser is used as a light source for the study of CdS emission, which generates a 388 nm laser pulse with frequency doubling of the fundamental 776 nm laser pulse having 80 MHz repetition rate by a second harmonic generation (SHG) technique. The emitting photons from CdS are collected by a streak camera. The spectrum of diffuse reflectance infrared Fourier transform (DRIFT) is acquired using a Bio-Rad FTS-45 spectrometer with a liquid N_2_-cooled MCT detector as collected at the 8 cm^–1^ resolution using a diffuse reflectance accessory of Spectra-Tech Collector. The sample is held in the sample cup of a Spectra-Tech high temperature environment chamber (HTEC) that could be resistively heated to 1000 K and the gas in the chamber evacuates to 10 μTorr. The structure and chemical composition of materials are analyzed with a field emission scanning electron microscope (LEO 1550 VP FESEM) that is equipped with an energy dispersive spectrometer (EDS). The XPS is obtained with an M-probe surface spectrometer (VG Instruments) using monochromatic Al Kα X-rays (1486.6 eV). The photocatalysis is performed by using a collimated output of a high-pressure 500 W Hg-Xe arc lamp as a light source in combination with a 400 nm cutoff filter. The evolved H_2_ is analyzed using gas chromatography (HPG1800A) with a thermal conductivity detector (TCD).

## 3 Results and Discussion

### 3.1 Structural Properties of Stoichiometric and Nonstoichiometric KNbO_3_

The KNbO_3_ structures synthesized at the 1:1 and 1:1.1 mole ratios of K_2_CO_3_ to Nb_2_O_5_ are identified by analysis of the XRD pattern, as shown in Figure S1. The stoichiometric KNbO_3_ (1:1) indicates the orthorhombic KNbO_3_ structure. The deposit process of Ni and CdS to KNbO_3_ (1:1) does not affect the KNbO_3_ skeletal structure as it indicates an identical XRD pattern. On the other hand, the nonstoichiometric structure of KNbO_3_ (1:1.1) generates a flawed KNbO_3_ structure, as shown in the characteristic layered structural peaks of K_4_Nb_6_O_17_·3H_2_O at 10, 28, 41, and 47, whose peak intensity is affected by the amount of Ni deposited on the surface of KNbO_3_ (1:1.1).

**Figure 1:**
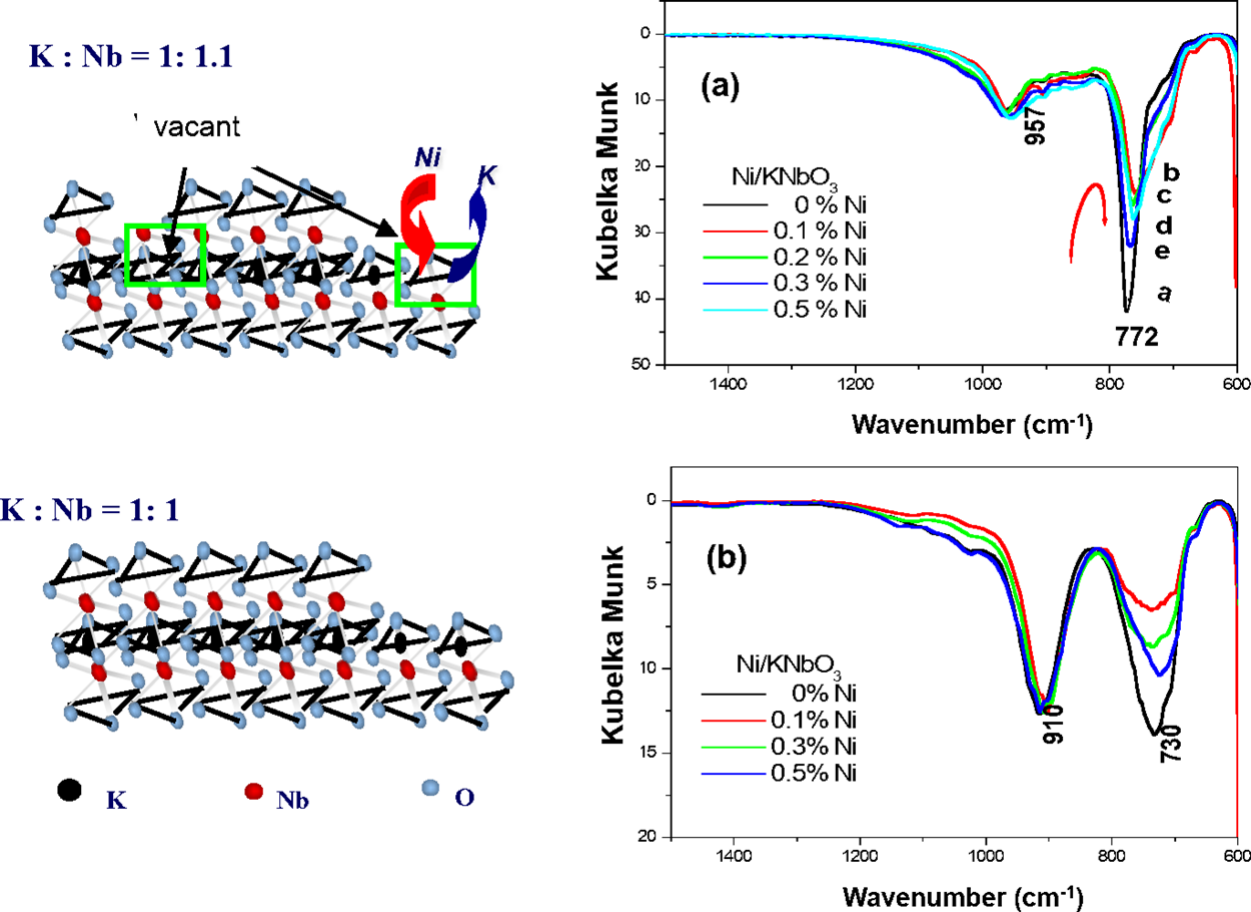
IR spectra of the nonstoichiometric (a) and the stoichiometric (b) structure of KNbO_3_ corresponding to Ni deposit amounts. The significant spectral change is obtained with the bridged O–Nb–O vibrational mode at 772 and 730 cm^–1^ for each of nonstoichiometric and stoichiometric KNbO_3_ structures.

Figure 1 exhibits the comparative IR spectra of two KNbO_3_ structures corresponding to Ni deposit amounts. The nonstoichiometric KNbO_3_ (a) indicates to have a higher vibrational energy than that of the stoichiometric KNbO_3_ (b) as follows: the stretching modes of a nonbridged Nb–O and a bridged O–Nb–O are detected at 959 and 772 cm^–1^, respectively, for the nonstoichiometric KNbO_3_ structure (a), while the stoichiometric KNbO_3_ (b) indicates those at 910 and 730 cm^–1^ with similar intensity. An apparent spectral change is observed at the bridged O–Nb–O caused by a Ni^2+^ deposit, resulting in a decreased peak intensity. The Ni effect appears in different aspects depending on the structure. For example, the nonstoichiometric KNbO_3_ structure (a) exhibits the peak shift from 772 to 756 cm^–1^ with the broadening effect, indicating a decreased vibrational energy as Δ*E* = 16 cm^–1^. On the other hand, the Ni effect to the stoichiometric KNbO_3_ (b) exhibits a decreased peak intensity at 730 cm^–1^ without significant energy change. Since the nonstoichiometric KNbO_3_ supposedly creates the vacancy at the K^+^ site, Ni^2+^ may occupy the vacancy during the process of Ni deposit, resulting in asymmetric bond strength with the bridged O–Nb–O caused by a charge sharing of one oxygen with adjacent Ni^2+^ as we assumed. The most significant change is obtained with 0.1 wt % Ni and then restored peak intensity gradually in case the Ni amount exceeds 0.1 wt %. We assumed that it is due to a formed Ni cluster.

Hereafter, the nonstoichiometric KNbO_3_ (1:1.1) will be denoted as KNbO_3_ because no conflict exists anymore since the present study is carried out using KNbO_3_ (1:1.1) only.

### 3.2 Photophysical Properties with the Electronic Structure of CdS/Ni/KNbO_3_ Composites

**Figure 2:**
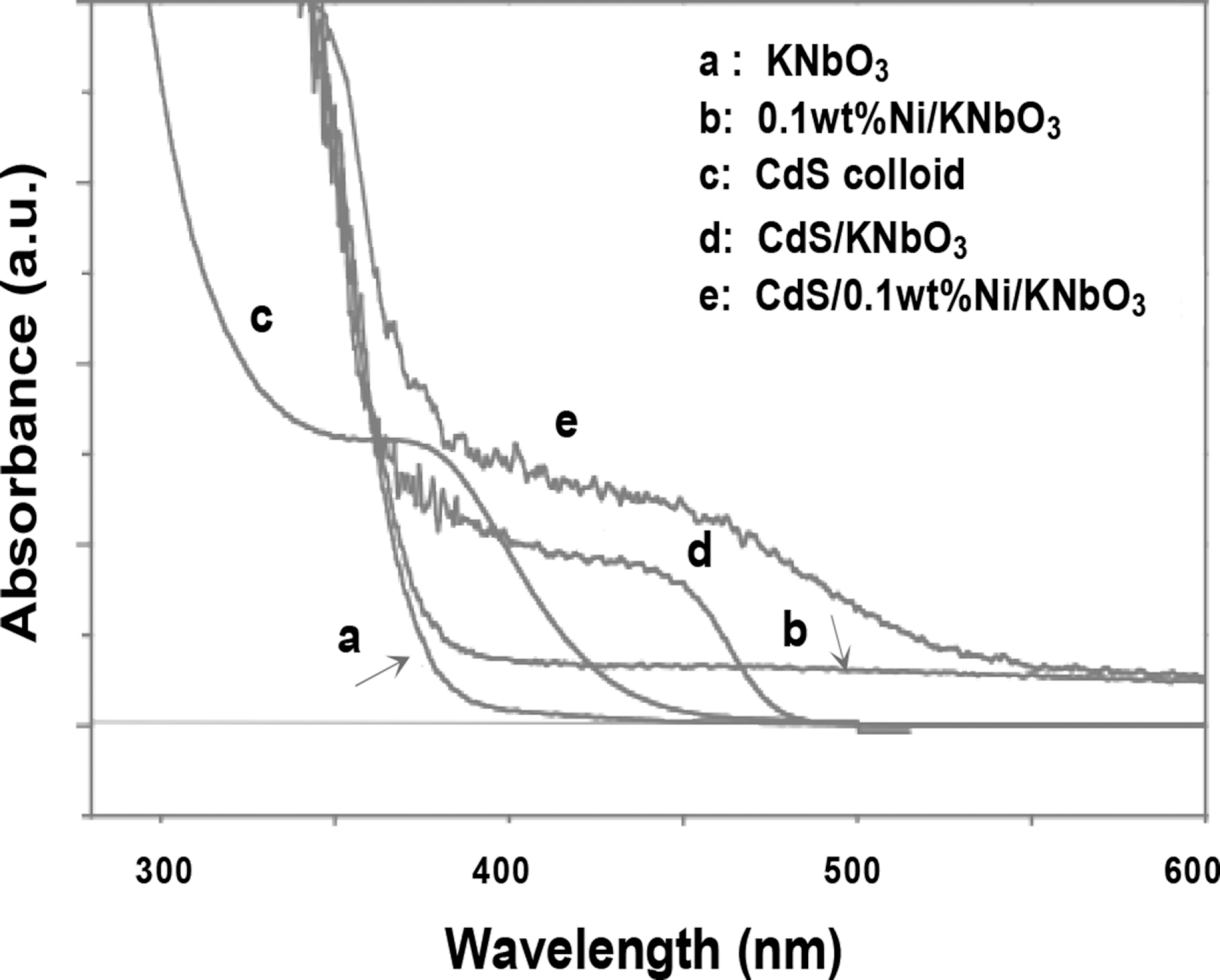
UV–vis diffuse reflectance spectra of KNbO_3_, Ni(0.1 wt %)/KNbO_3_, CdS/KNbO_3_, CdS/Ni(0.1 wt %)/KNbO_3_, and CdS colloids in ethanol.

The UV–vis diffuse reflectance spectra of KNbO_3_, Ni(0.1 wt %)/KNbO_3_, CdS/KNbO_3_, CdS/Ni(0.1 wt %)/KNbO_3_, and Q-size CdS colloids are exhibited in Figure 2. Based on the UV–vis absorption spectrum, the band gap of KNbO_3_ is obtained as 3.25 eV (±0.005) as calculated in Tauc’s method using a conversion factor between band gap energy (eV) and wavelength (nm). The 0.1 wt % Ni deposited on KNbO_3_ exhibits a weak absorption in the overall wavelength range between 380 and 750 nm. The CdS that deposited on the surface of KNbO_3_ is identified as nanosize particles (or clusters) by the TEM image as shown in Figure S2. The absorption band edge of CdS/KNbO_3_ and CdS/Ni(0.1 wt %)/KNbO_3_ is obtained at 478 and 540 nm, respectively. The red-shifted band edge with CdS/Ni(0.1 wt %)/KNbO_3_ is probably caused by a surface plasmon effect with the doped Ni, which causes coherent electron oscillation at the interface between CdS and KNbO_3_.

**Figure 3:**
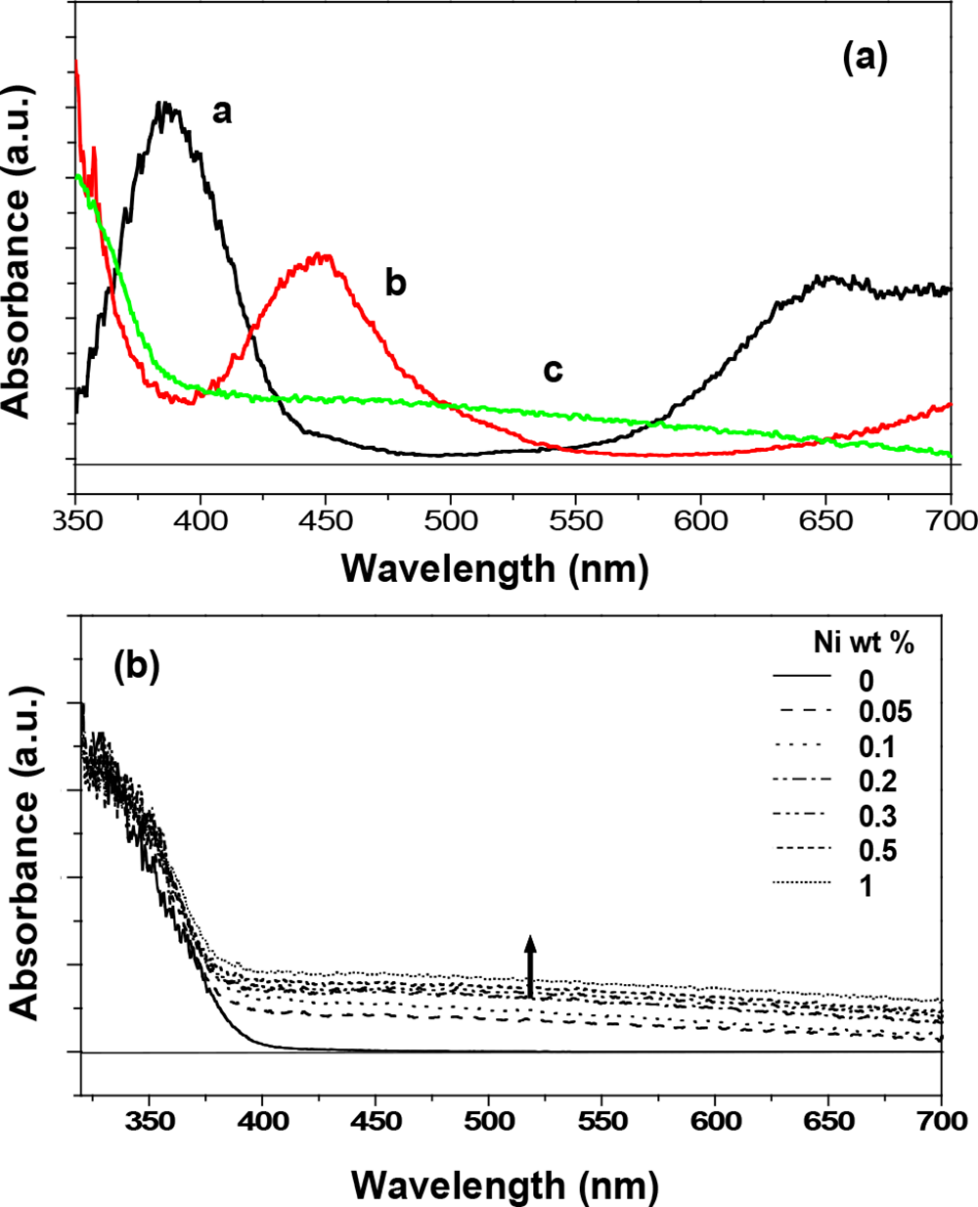
(a) UV–vis diffuse reflectance spectra of Ni species corresponding to the Ni deposit process: **a** (black), the absorption spectrum of Ni(NO_3_)_2_·6H_2_O powder as a precursor of Ni; **b** (red), the Ni^2+^ deposited on the surface of KNbO_3_ before H_2_ treatment; **c** (green), Ni/KNbO_3_ after H_2_ treatment at 500 °C for 2 h, followed by O_2_ treatment at 200 °C for 1 h. (b) UV–vis diffuse reflectance spectra of Ni/KNbO_3_ corresponding to the Ni amount of 0, 0.05, 0.1, 0.2, 0.3, 0.5, and 1.0 wt %, respectively.

The spectral analysis of the UV vis absorption in Figure 3 is performed corresponding to the Ni deposit process as follows: (a)-a is the absorption spectrum of Ni(NO_3_)_2_·6H_2_O as a Ni precursor; (a)-b, the absorption spectrum of Ni^2+^ that is physically adsorbed on the surface of KNbO_3_ as measured with the dried sample after immersion of KNbO_3_ in Ni(NO_3_)_2_ aqueous solution for 1 day; (a)-c, the absorption spectrum of Ni/KNbO_3_ after H_2_ treatment at 500 °C. In detail, the absorption peaks of Ni(NO_3_)_2_·6H_2_O at 390 and 655 nm in Figure 3(a)-a are attributed to the transition of ^3^A_2g_ (^3^F) → ^3^T_1g_ (^3^P) and ^3^A_2g_ (^3^F) → ^3^T_1g_ (^3^F), which are relevant to the transitions of divalent Ni ions, respectively.^18^ The deposit of Ni^2+^ on KNbO_3_ without H_2_ treatment indicates a spectral change that is band-shifted from 390 to 450 nm and 655 to 740 nm, respectively, as shown in Figure 3(a)-b (cut at 700 nm). The absorption bands at 450 and 740 nm are assigned to the transitions of ^3^A_2g_ (^3^F) → ^1^T_2g_ (^1^D) for the former and ^3^A_2g_ (^3^F) → ^1^E_1g_ for the latter, indicating a change of spin multiplicity from triplet to singlet that is basically addressed to the forbidden transition by a particular selection rule. However, the process of forbidden transition is allowed at a lower rate at a higher level of approximation (e.g., magnetic dipole, electric quadrupole).^19^ The change of Ni energy states is more likely a diffusion of Ni^2+^ into KNbO_3_, resulting in more stabilized Ni energy states caused by a charge transfer between Ni^2+^ and KNbO_3_ structure rather than the formation of NiO.^20,21^ The absorption spectrum of Ni^2+^ fades by a treated hydrogen at 500 °C. Instead, weak and broad absorption appears in the wavelength range between 380 and 700 nm, as shown in Figure 3(a)-c. This phenomenon is surely due to the reduction of Ni^2+^ to Ni^0^, and the weak and broad absorption is probably caused by a localized surface plasmon effect. Figure 3(b) evidently exhibits the increased surface plasmon absorption corresponding to an increased Ni amount.^22^

KNbO_3_ and Ni/KNbO_3_ are examined to be hardly active for photocatalytic H_2_ generation even under UV + visible light irradiation (λ ≥ 320 nm). According to Neumann et al., the cubic KNbO_3_ has a *d*^5^ electron configuration for Nb and consists of the valence band having a strong *d*-band character due to the evidential hybridization with O 2*p* and the conduction band with unoccupied Nb 4*d* states in the study of the density of states (DOS).^17^ On the other hand, Duan et al. revealed that the bottom of the conduction band is mostly derived from Nb 4*d* (LUMO) while the top of the valence band dominantly consists of O 2*p* (HOMO), indicating that the band gap energy varied from 3.1 to 3.8 eV (vs NHE) depending on the phase of KNbO_3_.^6,16,17,23^ The potential of valence and conduction bands for the orthorhombic KNbO_3_ is determined in between −0.26∼0 eV for CB and 3∼3.26 eV for VB (vs NHE at pH = 0) from the band gap energy of ca. ∼3.26 eV, which is insufficient potential to reduce H^+^ to H_2_ (refer to Figure 6).^14,23−25^

On the other hand, the hybrid composites, CdS/KNbO_3_ and CdS/Ni/KNbO_3_, exhibit fine photocatalytic activity for H_2_ production with a benefit of band gap engineering with CdS consisting of the band edges that enable abundant visible light absorption. Indeed, the potential of conduction and valence bands of CdS is higher than that of KNbO_3_ due to the higher energy states of Cd 4*d* and S 3*p* than those of Nb 4*d* and O 2*p*, respectively. Consequently, the CdS/KNbO_3_ composite consisting of a different band gap and potential energy has a big advantage for the photocatalysis with efficient charge separation between CdS and KNbO_3_ as constructed in a Z-schematic photocatalysis system. As a result, the significant enhancement of H_2_ production is obtained with CdS/Ni/KNbO_3_ due to the crucial role of Ni for efficient charge/electron transfer at the interface between CdS and KNbO_3_, which resulted in a retardation/prevention of electron–hole recombination.^24^

**Figure 4:**
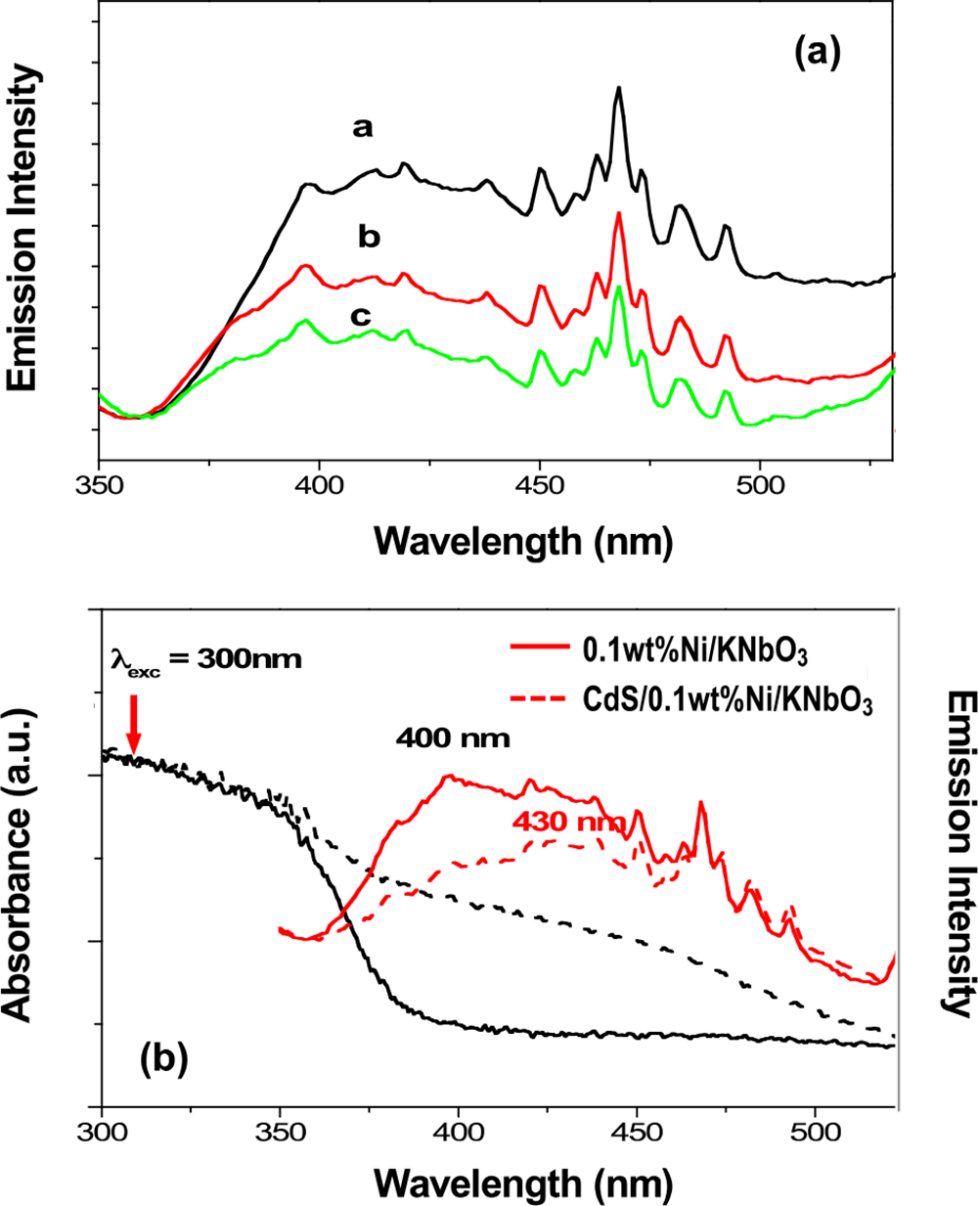
(a) Emission spectra of KNbO_3_ corresponding to the Ni deposit amount: a, 0%; b, 0.1 wt %; c, 0.3 wt %. (b) Comparative absorption (black) and emission spectra (red) of Ni(0.1 wt %)/KNbO_3_ (solid) and CdS/Ni(0.1 wt %)/KNbO_3_ (dot). All emission spectrum is obtained with 300 nm light excitation at the room temperature.

The mechanism of the internal photo-induced charge/electron transfer is examined with a spectral analysis of the KNbO_3_ emission corresponding to Ni and CdS deposit amounts. Figure 4(a) shows the emission spectra of KNbO_3_ corresponding to the Ni deposit amounts of 0, 0.1, and 0.3 wt %, respectively. KNbO_3_ indicates a broad emission around 400 nm with strong vibronic structural bands in the wavelength range between 450 and 500 nm, whose energy spacing is between 300 and 430 cm^–1^.^26^ The spectral change is detected with a Ni deposit resulted in a decreased emission intensity depending on Ni amounts, indicating an electron/charge transfer reasonably from KNbO_3_ to Ni. Figure 4(b) exhibits the KNbO_3_ emission spectra obtained with Ni(0.1 wt %)/KNbO_3_ comparing with and without CdS deposits. It indicates a spectral change with the CdS deposit, which quenches the emission around 400 nm where the CdS absorption occurred, as shown likely by a peak shifting from 400 to 430 nm. It is more likely caused by a charge/electron transfer to the deposited CdS. On the other hand, the conduction band of CdS is estimated as −0.55 eV vs NHE, and it is about −0.29 ∼ −0.55 eV higher potential than that of KNbO_3_. Although CdS may not be suitable energetically to accept electrons directly from CB of KNbO_3_, it would be possible to apply an indirect charge/electron transfer via Ni as an intermediator. Hence, we conclude that the quenched KNbO_3_ emission is caused by a charge/electron transfer to CdS via Ni.^27^ In detail, the photoexcited electrons by a 300 nm light irradiation on the Ni/KNbO_3_ are rapidly relaxed to the emissive states of KNbO_3_, and some of them might transfer to a doped Ni as shown by quenched emission corresponding to Ni amounts. In case of the CdS/Ni(0.1 wt %)/KNbO_3_ composite, the quenched KNbO_3_ emission at the energy region around 400 nm is detected, where CdS absorption occurred. We attributed it to a charge/electron transfer from KNbO_3_ to CdS via Ni.

**Figure 5:**
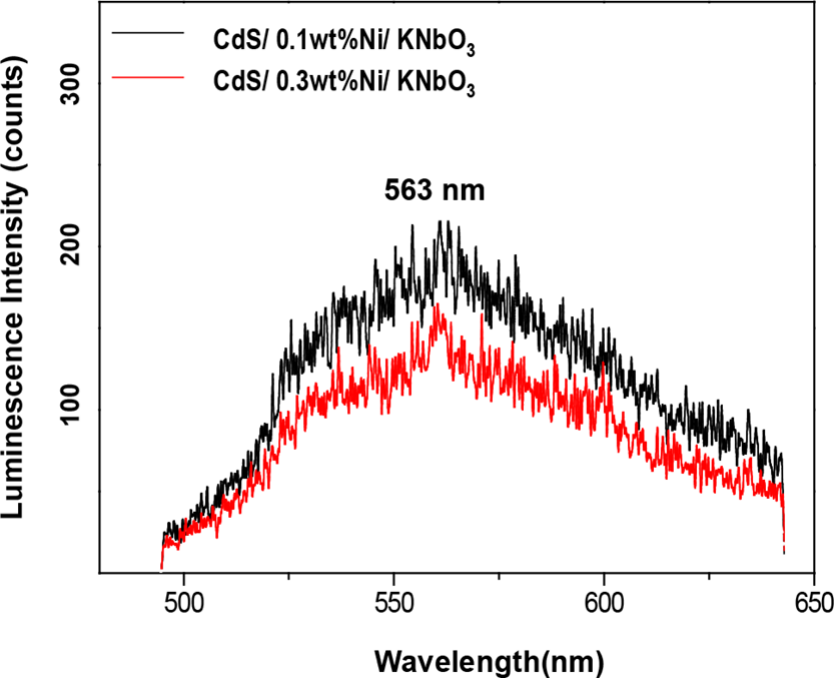
Emission spectra of CdS obtained with CdS/Ni/KNbO_3_ composites corresponding to Ni deposit amounts of 0.1 and 0.3 wt %, respectively, by 388 nm laser pulse photoexcitation.

The CdS emission is examined with CdS/Ni/KNbO_3_ composites corresponding to Ni amounts of 0.1 and 0.3 wt % (Figure 5). It is carried out using a 388 nm laser pulse as a light source and collected the emitted photons by using a streak camera because of the difficulty in detecting the CdS emission by using a conventional spectrofluorometer equipped with a lamp as a light source. The photoexcited electrons of CdS by a 388 nm laser pulse undergo fast nonradiative relaxation to the CdS trap states, followed by a radiative relaxation from the trap states to the ground state, as detected by the emission around 563 nm.^24,27,28^ The CdS emission tends to decrease the intensity with an increased Ni amount, as shown in comparison of 0.1 and 0.3 wt % Ni deposited CdS/Ni/KNbO_3_ composites. Indeed, the intensity of the emission is correlated with the number of radiative electrons and their retention time at the emissive states. For instance, Kamat and Shanghavi reported the CdS emission that is quenched by inter-particle electron transfer to the Au cluster in the composite of the CdS/Au cluster, and the quenching effects greatly increase with the increased amount of the core Au cluster.^29^ As we detected an identical quenching effect for the CdS emission with the 0.3 wt % Ni deposited CdS/Ni/KNbO_3_ composite, it is most likely due to the inter-particle electron transfer from the CdS to Ni cluster that is identified in excess Ni amount than 0.1 wt % (refer to Figure 1).

The luminescence study for KNbO_3_ and CdS with CdS/Ni/KNbO_3_ composites leads us to convince the CdS/Ni/KNbO_3_ composite as an efficient light conversion system with a crucial role of Ni in the interface between CdS and KNbO_3_, exhibiting a photo-induced electron relay with efficient charge/electron transfer between KNbO_3_ and CdS via Ni.

**Figure 6:**
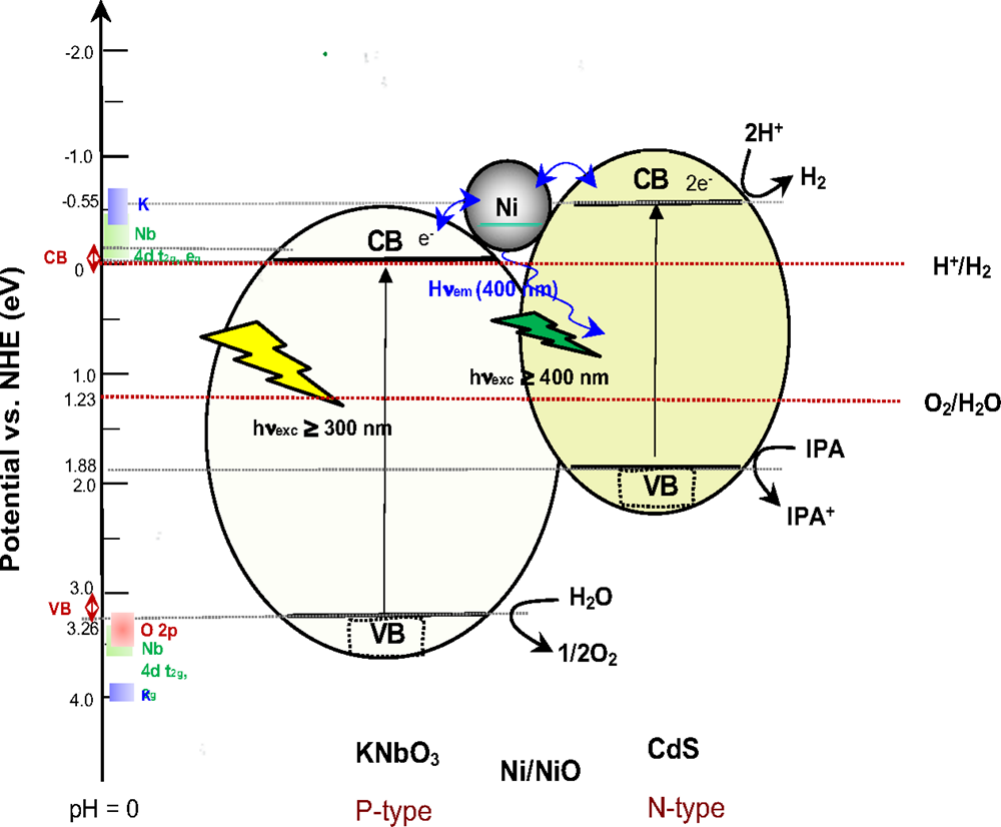
Suggested schematic diagram for the photo-induced electron relay in the CdS/Ni/KNbO_3_ composite as a Z-Scheme photocatalysis mechanism.

The suggested Z-schematic diagram for the photo-induced electron transfer mechanism with a photocatalytic H_2_ production is exhibited in Figure 6. In detail, the UV light irradiation (λ_exc_ < 380 nm) to CdS/Ni/KNbO_3_ composite induced the photo-generated electrons on the conduction band of KNbO_3_, followed by a nonradiative charge/electron transfer to CdS via Ni as seen in the quenched KNbO_3_ emission around 400 nm, promoting H_2_ generation at the CdS. Under visible light irradiation (λ_exc_ ≥ 400 nm), the H_2_ generates at the conduction band (CB) of CdS, while capturing the holes by IPA in the valence band (VB) of CdS. Under UV + visible light irradiation (λ ≥ 320 nm), it proceeds as a Z-schematic photocatalysis mechanism as a combination of two processes of KNbO_3_ and CdS.

### 3.3 Photocatalytic H_2_ Production with CdS/Ni/KNbO_3_ Composites

**Figure 7:**
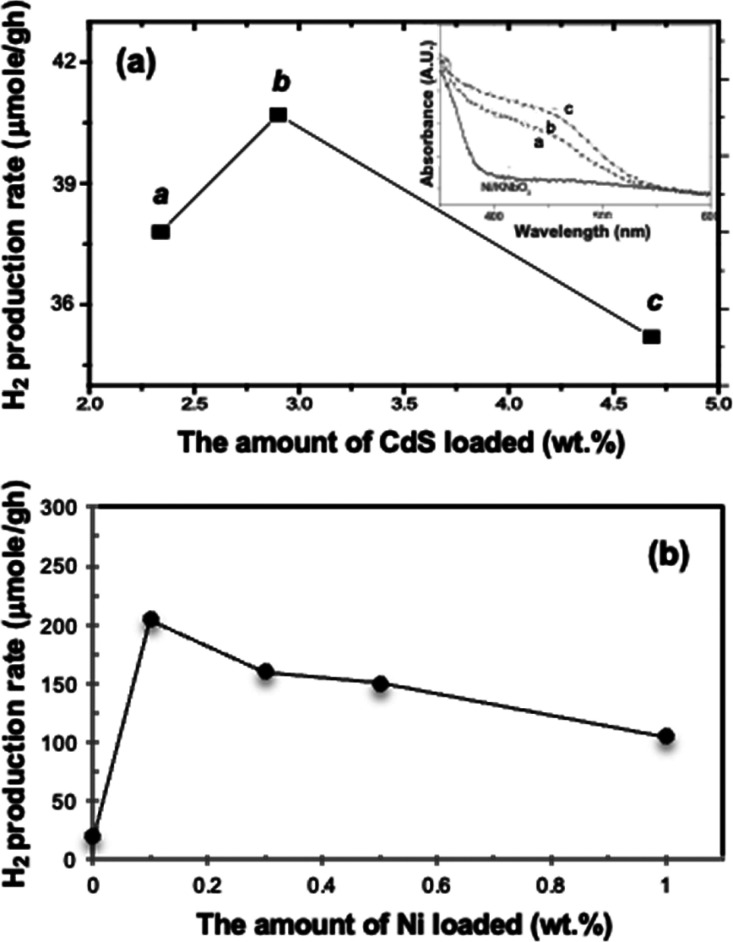
(a) Photocatalytic hydrogen production rates obtained with CdS/Ni/KNbO_3_ composites corresponding to the CdS amount. Inset is UV–vis diffuse reflectance spectra of CdS/Ni/KNbO_3_ corresponding to the CdS amount: **a**, 2.3 wt %; **b**, 2.9 wt %; **c**, 4.7 wt %. (b) Photocatalytic hydrogen production rates obtained after 2.9 wt % CdS deposit on Ni/KNbO_3_ that is 0, 0.1, 0.3, 0.5, and 1.0 wt % Ni deposited.

We investigate the photocatalytic H_2_ production with CdS/Ni/KNbO_3_ composites that is CdS and Ni amount dependent.^24^ Since the UV–vis diffuse reflectance spectra of CdS/Ni/KNbO_3_ composites indicate an enhanced absorbance in the visible region with an increased CdS amount, it is predicted that 4.7 wt % CdS composite is the best photocatalytic activity among the 2.3, 2.9, and 4.7 wt % CdS based on visible light utilization (Figure 7(a) inset). However, the photocatalytic H_2_ generation is in the order of 2.9 > 2.3 > 4.7 wt % CdS composite, as shown in Figure 7(a). Based on this, the photocatalytic hydrogen production is carried out further with 2.9 wt % CdS deposit to the Ni/KNbO_3_ that is 0, 0.1, 0.3, 0.5, 1.0 wt % Ni-deposited, respectively, and the result is exhibited in Figure 7(b). The best catalytic activity indicates 0.1 wt % Ni and 2.9 wt % CdS in the CdS/Ni/KNbO_3_ with a hydrogen production rate of 203.5 μmol/g h under visible light irradiation (λ_exc_ ≥ 400 nm).^24^ The photocatalytic activity of CdS/Ni/KNbO_3_ maintains the initial hydrogen generation rate as the first order kinetics for a whole photolysis period of 3 days.

On the other hand, the UV–vis diffuse reflectance spectra of CdS/KNbO_3_ and CdS/Ni/KNbO_3_ composite comparing before and after photocatalysis indicate an obvious spectral change, which is the CdS absorption edge shifted to a longer wavelength range for the recovery catalyst. It is most likely due to the aggregated CdS particles to stabilize the surface tension to avoid decomposition (Figure S3a,b). Alternatively, it might be due to the oxidized CdS during the recovery process as exposed to air.^30^ The photocatalytic H_2_ production carried out with the recovery CdS/Ni/KNbO_3_ dropped down to 60% of the initial activity (Figure S3c). The XPS spectra in Figure S3d evidently exhibit the electronic status of Cd, S, and Nb comparing before and after photocatalysis. The binding energy of the S 2*p* and Nb 3*p* electrons slightly increased for the recovery catalyst, indicating an oxidized status, while the electrons of Cd 3*d* indicate a reduced electronic status as obtained by the decreased binding energy.

We carried out the measurement of quantum yields, Φ, for the photocatalytic H_2_ production of CdS/Ni/KNbO_3_ composites. The apparent quantum yield, Φ, for H_2_ production is defined as follows:
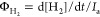
2where d[H_2_]/d*t* is the H_2_ production rate (mol s^–1^), and *I*_a_ is the number of absorbed photons in units of Einstein s^–1^ from the incident photons flux (*I*_0_) to the system.

**Figure 8:**
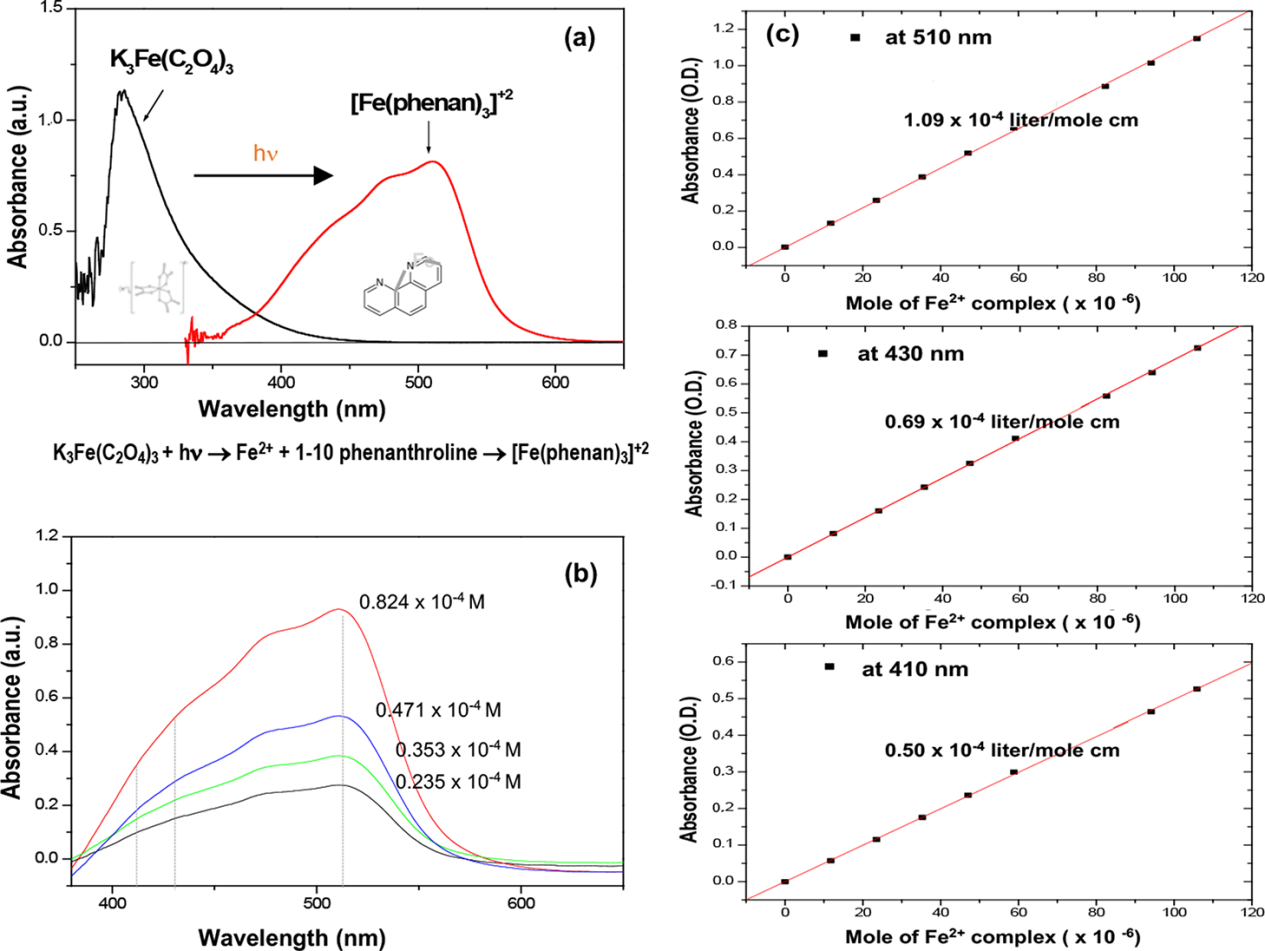
Optical properties of the potassium ferrioxalate actinometer: the potassium ferrioxalate, [K_2_F_2_(C_2_O_4_)_3_], is transformed to 1–10 phenanthroline, [Fe(phenan)_3_]^2+^, by photoirradiation. (a) Absorption spectra of the potassium ferrioxalate and 1–10 phenanthroline that are transformed by photoirradiation. (b) Absorbance of 1–10 phenanthroline, [Fe(phenan)_3_]^2+^, that is measured in various concentrations. (c) Molar attenuation coefficient (ε) of [Fe(phenan)_3_]^2+^ at the wavelength (λ) of 410, 430, and 510 nm, respectively.

The incident photons flux (*I*_0_) is obtained by using the potassium ferrioxalate [K_2_F_2_(C_2_O_4_)_3_] actinometer with the experimental setup for the photocatalytic hydrogen production reaction.^31,32^ The potassium ferrioxalate [K_2_F_2_(C_2_O_4_)_3_] is transformed to 1–10 phenanthroline [Fe(phenan)_3_]^2+^ during photoirradiation, as shown by the optical properties of an actinometer in Figure 8(a). The molar attenuation coefficient of 1–10 phenanthroline [Fe(phenan)_3_]^2+^ is determined at 410, 430, and 510 nm, respectively, with the absorption spectra obtained in the various concentrations, as shown in Figure 8b,c. The comparable molar attenuation coefficients of CdS colloid, K_3_Fe(C_2_O_4_)_3_, and 1–10 phenanthroline [Fe(phenan)_3_]^2+^ are obtained in the common wavelengths as listed in Table 1. As a result, the incident light flux to the photoreactor is defined as *I*_0_ = 1.27 × 10^17^ quanta/s for visible light (λ ≥ 400 nm) as it is obtained in the absence of photocatalysts.

**Table 1:** Comparison of the Molar Attenuation Coefficient (ε) of CdS Colloid, K_3_Fe(C_2_O_4_)_3_, and 1–10 Phenanthroline, [Fe(Phenan)_3_]^2+^, that Is Obtained at the Common Wavelengths (λ)

λ (nm)	CdS colloid (ε = *Α l*/mol cm)	K_3_Fe(C_2_O_4_)_3_ (ε = *A l*/mol cm)	[Fe(phenan)_3_]^2+^ (ε = *A l*/mol cm)
405	7.56 × 10^–2^	2.06 × 10^–2^	
410	7.06 × 10^–2^	1.67 × 10^–2^	0.5 × 10^–4^ (0.51)^a^
430	5.04 × 10^–2^	6.6 × 10^–3^	0.686 × 10^–4^ (0.696)^a^
510	8.13 × 10^–3^		1.09 × 10^–4^ (1.11)^a^

a The reference for the (ε) value of [Fe(phenan)_3_] ^2+^.

**Figure 9:**
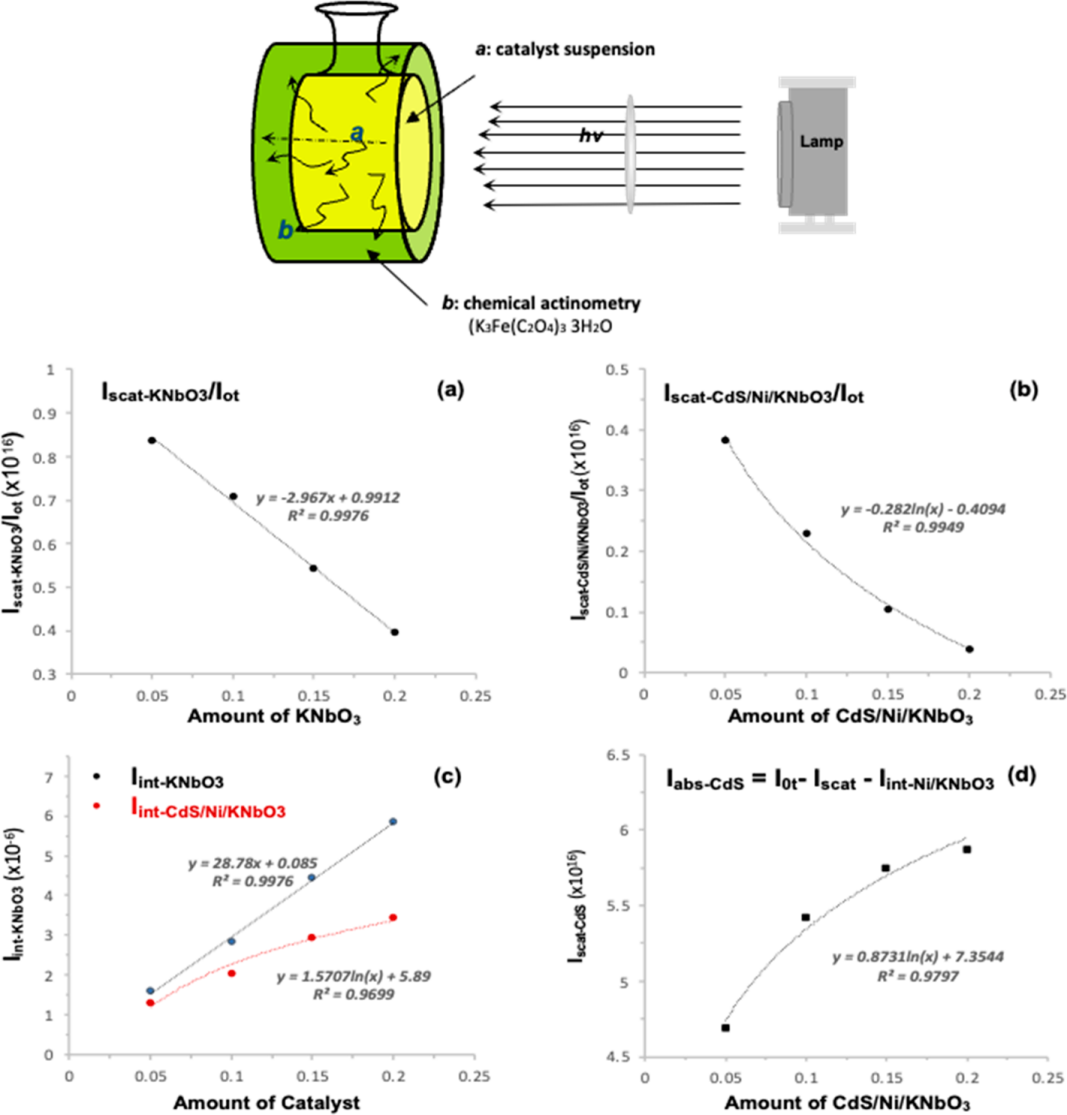
Scheme of the apparatus for a measurement of the scattered light flux from the catalyst (top). The bottom is the plots of the scattered light flux by KNbO_3_ (a) and CdS/Ni/KNbO_3_ (b) to the incident light flux (*I*_ot_); (c) plot of the internal energy of KNbO_3_ and CdS/Ni/KNbO_3_; (d) plot of the absorbed photon flux by CdS with CdS/Ni/KNbO_3_ corresponding to the catalyst amount of 0.05, 0.1, 0.15, and 0.2 g, respectively.

The CdS/Ni/KNbO_3_ composites have the particle size around 1 μm. The scattered photon flux (*I*_scat_) is calculated by a ferrioxalate [K_3_Fe(C_2_O_4_)_3_] actinometry with the specially designed photoreactor, which enables to detect a back- and side-scattered light flux that passed through the photolysis reactor (refer to the apparatus in Figure 9).

The scattered light flux from KNbO_3_ and CdS/Ni/KNbO_3_ is obtained with visible light irradiation (λ ≥ 400 nm) corresponding to the catalyst amount of 0.05, 0.10, 0.15, and 0.20 g, respectively, and presented as the plots of *I*_scat_/*I*_ot_ in Figure 9a,b.

Since there is no light absorbance by KNbO_3_ in the visible light, the difference between the incident (*I*_ot_) and the scattered light flux (*I*_scat_) is considered as the internal energy of KNbO_3_ that is consumed as molecular vibrational and rotational modes while showing multiple light scattering among the particles as shown in the equation below.
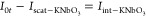
3

On the other hand, the scattered light flux (*I*_scat_) by CdS/Ni/KNbO_3_ might be a subtraction of the absorbed light flux by CdS nanoparticles (*I*_abs-CdS_) and the internal energy of Ni/KNbO_3_ (*I*_int – Ni/KNbO_3__) from the incident light flux (I_ot_). The consumed photons as an internal energy of Ni/KNbO_3_ (*I*_int – Ni/KNbO_3__) must be less than that by itself of KNbO_3_ because a limited surface area of Ni/KNbO_3_ is exposed to the incident light flux (*I*_ot_) due to the CdS nanoparticles deposited on the surface. The impact may increase according to the increased catalyst amount of CdS/Ni/KNbO_3_. The internal energy of Ni/KNbO_3_ is estimated to be a logarithmic function corresponding to the increased catalyst amount while the KNbO_3_ indicates to be a linear function, as shown in Figure 9(c). The absorbed photons by CdS nanoparticles (*I*_abs-CdS_), which are evaluated as effective and utilizable photons to the photocatalytic H_2_ production, are calculated corresponding to the catalyst amount with the equation below and presented the plot in Figure 9(d).
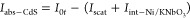
4

Since the scattered light flux *I*_scat_ is dependent on the catalyst amounts corresponding to the incident light flux (*I*_0_), we defined the index for *I*_abs-CdS_ of CdS/Ni/KNbO_3_ as the portion of *I*_abs-CdS_ to *I*_0*t*_. The index is applicable to the designated amounts of CdS/Ni/KNbO_3_ as listed in Table 2. For example, the index with 0.2 g catalyst amount is defined as 0.605, which means that 0.2 g of CdS/Ni/KNbO_3_ estimates to absorb and utilize approximately 60.5% of the incident photon flux (*I*_0_) for the photocatalytic molecular hydrogen production. As a result, the absorbed light flux (*I*_a_) by 0.2 g of CdS/Ni/KNbO_3_ including 2.9 wt % CdS and 0.1 wt % Ni indicates approximately 7.68 × 10^16^ quanta/s after applying the index of 0.605 to the incident light flux (*I*_0_) of 1.27 × 10^17^ (λ ≥ 400 nm) to the photoreactor. The corresponding quantum yield for the photocatalytic hydrogen generation of ∼40 μmol/h under visible light (λ ≥ 400 nm) is determined as 8.8% in 30% IPA aqueous solution from the equation, Φ_H_2__ = d[H_2_]/d*t* × (6.023 × 10^23^)/*I*_a_.

**Table 2:** Scattered (*I*_scat_) and the Absorbed Photon Flux (*I*_a_) Corresponding to KNbO_3_ and CdS/Ni/KNbO_3_ Amount with the Portion to the Incident Light Flux as *I*_scat_/*I*_ot_ (%) and *I*_a_/*I*_ot_^a^^,^^b^

		under\vis (λ > 400 nm) (quanta/s)											
		*I*_scat_				*I*_scat_/*I*_0*t*_ (%)				*I*_a_/*I*_0*t*_			
catalyst\(amount)		0.05 g	0.10 g	0.15 g	0.20 g	0.05 g	0.10 g	0.15 g	0.20 g	0.05 g	0.10 g	0.15 g	0.20 g
KNbO_3_	*I*_scat_	8.1 × 10^16^	6.87 × 10^16^	5.26 × 10^16^	3.84 × 10^16^	83.5	70.8	54.2	39.6				
	*I*_int – KNbO_3__									1.6 × 10^16^ (16.5%)	2.83 × 10^16^ (29.2%)	4.44 × 10^16^ (45.8%)	5.86 × 10^16^ (60.4%)
CdS/Ni/KNbO_3_	*I*_scat_	3.71 × 10^16^	2.22 × 10^16^	1.01 × 10^16^	3.70 × 10^15^	38.2	22.9	10.4	3.8				
	*I*_int – Ni/KNbO_3__									1.3 × 10^16^	2.03 × 10^16^	2.94 × 10^16^	3.46 × 10^16^
	*I*_abs-CdS_									4.69 × 10^16^ (48.4%)	5.45 × 10^16^ (56.2%)	5.75 × 10^16^ (59.3%)	5.87 × 10^16^ (60.5%)
							index (*I*_a_)^c^ of CdS/Ni/KNbO_3_			0.484	0.562	0.593	0.605

a The portion of the CdS absorbed photons to the incident light flux (*I*_abs-CdS_/*I*_0*t*_) is determined as the index (*I*_a_)^c^, which is applicable to the designated CdS/Ni/KNbO_3_ catalyst amount for a quantum yield measurement. *I*_0*t*_ = 9.7 × 10^17^ quanta/s, when (1 – 10^–ε[*Α*]*l*^) = 1.

b *I*_scat_ by KNbO_3_ = *I*_scat – KNbO_3__ = *I*_0*t*_ – *I*_scat – KNbO_3__; *I*_abs – KNbO_3_(λ > 400nm)_ = *I*_int – KNbO_3__; *I*_scat_ by CdS/Ni/KNbO_3_ = *I*_scat – CdS/Ni/KNbO_3__ = *I*_0*t*_ – (*I*_int – Ni/KNbO_3__ + *I*_abs-CdS_); *I*_abs – CdS/Ni/KNbO_3_(λ > 400nm)_ = *I*_int – Ni/KNbO_3__ + *I*_abs-CdS_; *I*_abs-CdS_ = *I*_abs – CdS/Ni/KNbO_3_(λ > 400nm)_ – *I*_int – Ni/KNbO_3__; *I*_scat – CdS/Ni/KNbO_3__ = *I*_0*t*_ – (*I*_int – Ni/KNbO_3__ – *I*_abs-CdS_).

c Index *I*_a_ of CdS/Ni/KNbO_3_ is the portion of *I*_abs-CdS_ to *I*_0*t*_ corresponding to the catalyst amount.

## 4 Conclusions

In this study, we investigate the structural properties of KNbO_3_ comparing the stoichiometric and nonstoichiometric KNbO_3_ structure corresponding to Ni deposit, and the photophysical properties and photocatalytic activity of CdS/Ni/KNbO_3_ composites as well.

The FTIR spectral analysis comparing the stoichiometric (1:1) and nonstoichiometric (1:1.1) KNbO_3_ structure reveals the different aspects of the deposited Ni effects corresponding to the structure. The nonstoichiometric structure of KNbO_3_(1:1.1) generates the defects at the K^+^ site, which is Ni-occupied during its deposit process. It exhibits a tendency like a doped Ni into KNbO_3_ up to 0.1 wt % of Ni and then forms a Ni cluster in case the amount exceeds 0.1 wt %. The photophysical properties of CdS/Ni/KNbO_3_ examined with UV–vis absorption and luminescence spectral analysis demonstrates the CdS/Ni/KNbO_3_ composites to be efficient light conversion caused by efficient charge/electron transfer between KNbO_3_ and CdS via doped Ni.

The photocatalytic activity of CdS/Ni/KNbO_3_ exhibits a CdS and Ni amount dependency. The best photocatalytic activity for H_2_ generation is obtained with 0.1 wt % Ni and 2.9 wt % CdS composite as it gradually declines with the excess Ni amounts than 0.1 wt % caused by a formed Ni cluster.

## Supporting Information Available

Comparative XRD pattern of the stoichiometric KNbO_3_ (1:1) and the nonstoichiometric KNbO_3_ (1:1.1) structure; TEM image of CdS nanoparticles distributed on the surface of KNbO_3_; comparative UV–vis absorption and XPS spectra of CdS/Ni/KNbO_3_ composites before and after 24 h photocatalysis with the photocatalytic H_2_ production rates (PDF)
