# Supplementary material for: Spectroscopic Study on CdS/Ni/KNbO3: Confirming Ni Effect to Photocatalytic Activity
Source: ACS Omega. 2023 Sep 11;8(38):35173–82. doi: 10.1021/acsomega.3c04829 (PMC10536861; doi:10.1021/acsomega.3c04829)
Supplement: Supplementary file 1 — ao3c04829_si_001.pdf [file ao3c04829_si_001.pdf]

Supporting Information

Spectroscopic Study on CdS/Ni/$KNbO_3$: Confirming Ni effect to Photocatalytic Activity

Su Young Ryu*, Tai Kyu Lee, and Michael R. Hoffmann*

Environmental Science & Engineering

Linde Laboratory

California Institute of Technology

Pasadena, CA 91125, USA

Corresponding authors E-mail: syryu7@gmail.com and mrh@caltech.edu

Tel: +1-626-395-4391

Fax: +1-626-395-2940

Contents:

Figure S1. The comparative XRD pattern of the stoichiometric $KNbO_3$ (1:1) (a) and the non-stoichiometric $KNbO_3$ (1:1.1) structure (b), indicating the orthorhombic $KNbO_3$ structure having an identical XRD pattern after Ni and CdS deposit to $KNbO_3$ (1:1), while the non-stoichiometric $KNbO_3$ (1:1.1) generates the flawed structure of $KNbO_3$ as shown the peaks of layered structure of $K_4Nb_6O_{17}$ (*) at 10, 28, 41, and 47 (2θ), whose the peak intensity is affected by the Ni amount deposited to the surface of $KNbO_3$ (1:1.1).

Figure S2. TEM image of CdS nanoparticles distributed on the surface of $KNbO_3$. (a) the well dispersed CdS nano-particles (~ 3 nm) and the aggregated forms of CdS (b).

Figure S3. The UV-vis absorption spectra of CdS (2.9 wt.%)/Ni(0.1 wt.%)/$KNbO_3$ (a) and CdS (2.9 wt.%)/$KNbO_3$ (b) measured before (black) and after (red) 24 h photocatalysis. (c) the obtained photocatalytic H_2 production rates. (d) XPS spectra of Cd, S, and Nb as obtained with CdS (2.9 wt.%)/Ni(0.1 wt.%)/$KNbO_3$ before (black) and after (red) photocatalysis.

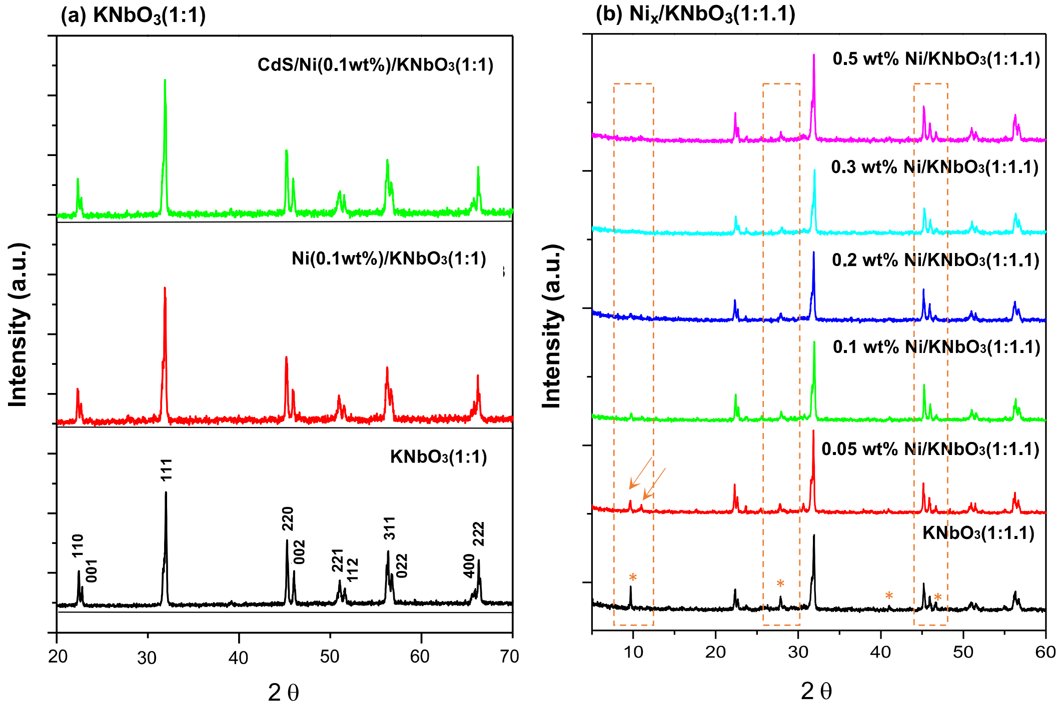

Figure S1. The comparative XRD pattern of the stoichiometric $KNbO_3$ (1:1) (a) and the non-stoichiometric $KNbO_3$ (1:1.1) structure (b), indicating the orthorhombic $KNbO_3$ structure having an identical XRD pattern after Ni and CdS deposit to $KNbO_3$ (1:1), while the non-stoichiometric $KNbO_3$ (1:1.1) generates the flawed structure of $KNbO_3$ as shown the peaks of layered structure of $K_4Nb_6O_{17}$ (*) at ~10, 28, 41, and 47 (2θ), whose the peak intensity is affected by the Ni amount deposited to the surface of $KNbO_3$ (1:1.1).

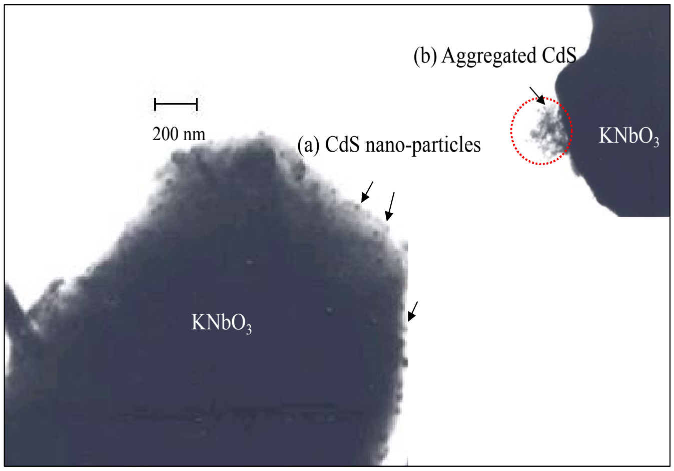

Figure S2. TEM image of CdS nanoparticles distributed on the surface of $KNbO_3$. (a) the well dispersed CdS nano-particles (~ 3 nm) and the aggregated forms of CdS (b).

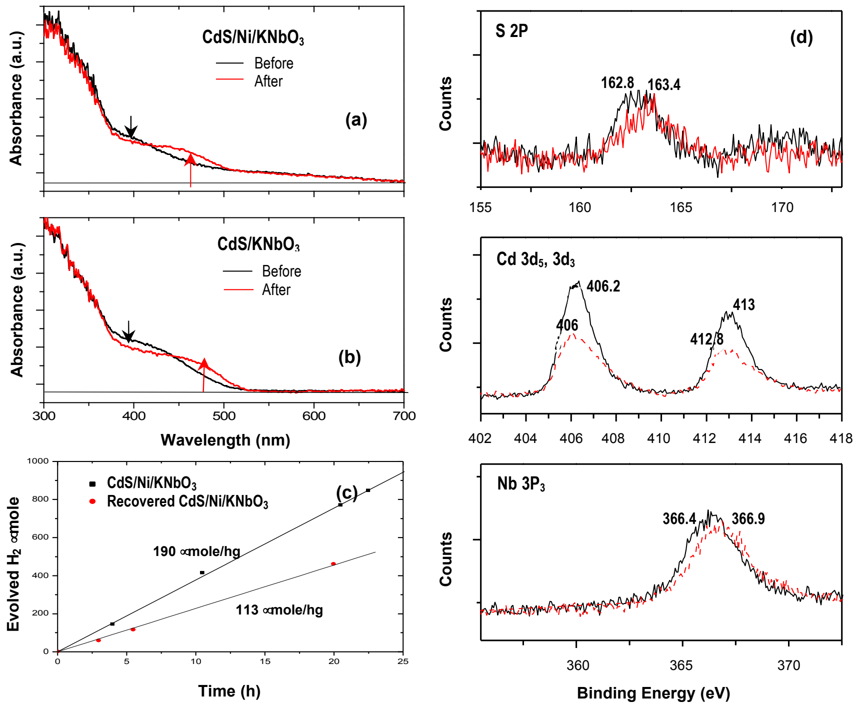

Figure S3. The UV-vis absorption spectra of CdS (2.9 wt.%)/Ni(0.1 wt.%)/$KNbO_3$ (a) and CdS (2.9 wt.%)/$KNbO_3$ (b) measured before (black) and after (red) 24 h photocatalysis. (c) the obtained photocatalytic H_2 production rates. (d) XPS spectra of Cd, S, and Nb as obtained with CdS (2.9 wt.%)/Ni(0.1 wt.%)/$KNbO_3$ before (black) and after (red) photocatalysis.
